# Organic anion transporting polypeptide 1B3 can form homo- and hetero-oligomers

**DOI:** 10.1371/journal.pone.0180257

**Published:** 2017-06-23

**Authors:** Yuchen Zhang, Kelli H. Boxberger, Bruno Hagenbuch

**Affiliations:** Department of Pharmacology, Toxicology and Therapeutics, the University of Kansas Medical Center, Kansas City, Kansas, United States of America; University of Cambridge, UNITED KINGDOM

## Abstract

OATP1B3 is a 12 transmembrane domain protein expressed at the basolateral membrane of human hepatocytes where it mediates the uptake of numerous drugs and endogenous compounds. Previous western blot results suggest the formation of OATP1B3 multimers. In order to better understand the function of OATP1B3 under normal physiological conditions, we investigated its oligomerization status. We transiently transfected OATP1B3 with a C-terminal His-, FLAG- or HA-tag in HEK293 cells and used co-immunoprecipitation and a Proximity Ligation Assay to detect interactions between the different constructs. All three constructs retained similar transport rates as wild-type OATP1B3. Immunofluorescence experiments indicated that in contrast to wild-type, His- and FLAG-tagged OATP1B3, where the C-terminal end is on the cytoplasmic side of the membrane, the C-terminal end of HA-tagged OATP1B3 is extracellular. After cross-linking, anti-FLAG antibodies were able to pull down FLAG-tagged OATP1B3 (positive control) and co-transfected His- or HA-tagged OATP1B3, demonstrating the formation of homo-oligomers and suggesting that the C-terminal part is not involved in oligomer formation. We confirmed co-localization of His- and FLAG-tagged OATP1B3 in transfected HEK293 cells with the Proximity Ligation Assay. Transport studies with a non-functional OATP1B3 mutant suggest that the individual subunits and not the whole oligomer are the functional units in the homo-oligomers. In addition, we also detected OATP1B3-FLAG co-localization with OATP1B1-His or NTCP-His, suggesting that OATP1B3 also hetero-oligomerizes with other transport proteins. Using the Proximity Ligation Assay with transporter specific antibodies, we demonstrate close association of OATP1B3 with NTCP in frozen human liver tissue. These findings demonstrate that OATP1B3 can form homo- and hetero-oligomers and suggest a potential co-regulation of the involved transporters.

## Introduction

Organic anion transporting polypeptide (OATP) 1B3 is a multispecific drug uptake transporter belonging to the *SLCO* superfamily [1]. Under normal physiological conditions, OATP1B3 and the closely related OATP1B1 are selectively expressed at the sinusoidal membrane of human hepatocytes [2]. The two drug transporters are responsible for the uptake of numerous endogenous compounds, such as bile acids and hormones, and many xenobiotics, including various drugs, into hepatocytes [3]. In addition, conjugated bile acids, including taurocholate or glycocholate, are transported into human hepatocytes via Na^+^/Taurocholate Cotransporting Polypeptide (NTCP) [4] which has recently also been identified as the receptor for human hepatitis B and D virus [5].

Drug uptake transporters contribute to drug metabolism like the cytochrome P450 drug-metabolizing enzymes, and both can be modulated at different levels [6, 7]. The most direct way is to modulate OATP-mediated drug uptake with compounds that either inhibit or stimulate the transporter. Several studies have demonstrated that inhibition of OATP1B1 and/or OATP1B3 can lead to drug-drug-interactions [8]. For example, the rifampicin—bosentan interaction that was observed in healthy subjects [9] or the interaction between bosentan and sildenafil [10] could both be explained by direct inhibition of OATP1B1- and OATP1B3-mediated bosentan uptake by rifampicin and sildenafil [11]. In addition to inhibition, direct stimulation of OATP-mediated uptake has also been reported. OATP1B3-mediated uptake of estradiol-17β-glucuronide (E17βG) can be stimulated by clotrimazole [12] or a substituted quercetin [13]; OATP1B3-mediated uptake of estrone-3-sulfate (E3S) is stimulated by the green tea catechin epigallocatechin gallate [14]; and OATP1B1- or OATP1B3-mediated uptake of pravastatin is stimulated by NSAIDS like diclofenac or ibuprofen [15]. Importantly, these stimulatory effects increased the affinities of the substrates to the OATPs, indicating allosteric modulation. Besides direct interactions with the transport protein, the function of OATPs can also be regulated at the transcriptional, translational, or even post-translational level [6, 16, 17]. Furthermore, there is the potential for protein-protein interactions that could influence transporter function, similar to what has been shown for drug-metabolizing enzymes [18]. It has been demonstrated that some rat transporters expressed in hepatocytes, including NTCP [19] and OCT1, can form homo-oligomers [20]. In addition, a recent study showed that human OATP1B1 may form and function as oligomers [21]. However, no such studies have been reported so far for human OATP1B3. Therefore, the goal of the present study was to investigate whether human OATP1B3 could form homo- or hetero-oligomers and how such oligomerization would affect transport function.

## Materials and methods

### Materials

Human embryonic kidney (HEK293) cells were purchased from ATCC (Manassas, VA); the cells were routinely tested for the absence of mycoplasma using the MycoAlert™ Mycoplasma Detection Kit (Lonza, Hopkinton, MA). Frozen human liver was obtained through the Cell Isolation Core in the Department of Pharmacology, Toxicology and Therapeutics at the University of Kansas Medical Center, in accordance with a protocol approved by the Institutional Review Board of the University of Kansas Medical Center, from patients undergoing hepatic resection procedures or from liver donors. Radioactive compounds [^3^H]estrone-3-sulfate (E3S, 54.0 Ci/mmol) and [^3^H]estradiol-17β-glucuronide (E17βG, 49.8 Ci/mmol), were obtained from Perkin Elmer (Waltham, MA). The crosslinking reagent 3,3'-dithiobis(sulfosuccinimidyl propionate) (DTSSP) and Dynabeads® Protein G for immunoprecipitation were purchased from ThermoFisher (Waltham, MA). Primary antibodies were obtained from the following suppliers: rabbit anti-His antibody from Abcam (Cambridge, MA); mouse anti-HA antibody from LifeTein (Hillsborough, NJ); mouse anti-SLC22A1 (2C5) antibody from Novus (St. Charles, MO); mouse anti-His antibody from Qiagen (Hilden, Germany); goat anti-NTCP antibody from Santa Cruz Biotechnology (Dallas, TX); mouse anti-FLAG antibody and mouse anti-FLAG antibody conjugated to magnetic beads from Sigma-Aldrich (St. Louis, MO). The rabbit anti-OATP1B3 antibody (K28) was a generous gift from Dr. Bruno Stieger (University Hospital Zurich, Switzerland). Duolink Proximity Ligation Assay reagents were purchased from Sigma-Aldrich. All other chemicals and reagents were of analytical grade and were readily available from commercial sources.

### Generation of OATP1B3 with a FLAG- or HA-tag at the C-terminal end

To generate OATP1B3 with either a C-terminal FLAG- or HA-tag, the open reading frame (ORF) of OATP1B3 was amplified with the following primers using the expression plasmid encoding the His-tagged OATP1B3 [22] as a template: Forward, 5’ GCTAGCTAATACGACTCACTATAGGGACCATGGAC 3’; OATP1B3-FLAG reverse, 5’ GCGGCCGCTTACTTAT CGTCGTCATCCTTGTAATCGTTGGCAGCAGCATTGTCTTGCATGT 3’; OATP1B3-HA reverse, 5’ GCGGCCGCTTAAGCGTAATCTGGAACATCGTATGGGTAGTTGGCAGCAGCATTGTCTTGCATGT 3’. After amplification, the ORF of OATP1B3 with either a FLAG- or an HA-tag was inserted between the *NheI* and *XhoI* sites of the pcDNA5/FRT plasmid. The resulting constructs were sequenced to verify insertion of the desired tags.

### Cell culture and transient transfection

HEK293 cells were grown and transfected using Fugene HD (Active Motif, Carlsbad, CA) as previously described [23].

### Transport assays

[^3^H]E3S and [^3^H]E17βG uptake with HEK293 cells expressing the differently tagged OATP1B3 constructs was performed on 24-well plates 48 hours after transfection as previously described [24].

### Immunofluorescence

HEK293 cells were plated at a density of 90,000 cells/well on 4-well slides (ThermoFisher) and transfected with the respective expression plasmid. Forty-eight hours after transfection, the slides were fixed and permeabilized with 2% paraformaldehyde with or without 1% Triton X-100 for 10 minutes and blocked with 5% normal donkey serum for 1 hour at room temperature. Cells were then incubated at 4°C overnight with the respective monoclonal primary antibodies in 1% normal donkey serum in phosphate-buffered saline (PBS). After washing with PBS, slides were incubated with goat anti-mouse AlexaFluor 488 (ThermoFisher), as secondary antibody, diluted 1:1000 in 0.1% PBS-Tween for 1hour, and after a final wash, slides were mounted in Prolong Gold containing DAPI (ThermoFisher). For negative controls, the sections were incubated with secondary antibody only.

### Crosslinking and isolation of membrane fractions

HEK293 cells were plated at a density of 600,000 cells/well on 6-well plates (TPP, Switzerland) and transfected with the respective expression plasmids. After 48 hours of culture, the cells were washed with PBS and then incubated in PBS with or without 2mM DTSSP in PBS for crosslinking at 4°C for 2 hours. Crosslinking was stopped by incubating with 20mM Tris-HCl (pH 7.5) for 15 minutes. Cells were then washed with ice-cold PBS, and cold hypotonic homogenization solution (1mM NaCl, 5mM Tris-HCl pH 7.5) was added before cells were scraped off the plates. The cells were homogenized with 20 strokes using a glass-Teflon homogenizer, and the homogenates were centrifuged at 900 x g for 10 minutes. The resulting supernatants were centrifuged at 10,000 x g for 20 minutes and the pellets containing the membrane fractions were re-suspended in hypotonic homogenization solution and used for immunoprecipitation experiments.

### Immunoprecipitation

The isolated membrane fractions were solubilized in 0.1% NP-40 in PBS for 10 minutes on ice and centrifuged at 14,000 x g for 30 minutes to remove insoluble components. Then 5μL of anti-His antibody or 20μL of anti-FLAG antibody-conjugated beads were added and the solution was incubated at 4°C with end-over-end rotation for 2 days. For the anti-His antibody treated samples, 20μL of Dynabeads protein G were added after the first 24 hours of incubation. At the end of the incubation, the beads were separated with a magnet and washed with 0.1% NP-40 in PBS twice for 15 minutes each. The purified proteins were eluted with 1% TX-100 in 0.1M glycine (pH 2.5).

### Surface biotinylation

HEK293 cells were plated and transfected as described above for the crosslinking experiments. After 48 hours of culture, the cells were washed with PBS and incubated in PBS containing 1mg/mL EZ-Link NHS-SS-Biotin (ThermoFisher) at 4°C for 2 hours on a rocker. Cells were then washed and biotinylation was stopped by incubating cells with PBS containing 100mM glycine at 4°C for 30 minutes with rocking. After washing with ice-cold PBS, cells were subsequently lysed using lysis buffer (10mM Tris, 150mM NaCl, 1mM EDTA, 0.1% SDS, 1% Triton X-100, pH 7.5). Insoluble debris was removed by centrifugation at 14,000 x g for 30 mins. Pre-washed NeutrAvidin Agarose Resin (50 μL, ThermoFisher) was added to each sample and incubated with end-over-end rotation at room temperature for 2 hours. After washing with lysis buffer twice, surface proteins were eluted and collected by incubating with 1 X Laemmli buffer containing β-mercaptoethanol and protease inhibitors at room temperature for 30 minutes.

### Western blot analysis

SDS-PAGE was performed with a mini-protean Tetra system from Bio-Rad. Immunopreciptated proteins were mixed with 5 X Laemmli-buffer (300mM Tris-Cl pH 6.8, 10% SDS, 50% glycerol, 0.05% bromophenol blue and 12.5%β-mercaptoethanol) and separated on 4%-20% gradient SDS gels (Expedeon, San Diego, CA) for 2 hours. After SDS-PAGE, separated proteins were transferred to PVDF membranes using the Trans-Blot Turbo Transfer system from Bio-Rad. The membranes were then blocked with 5% non-fat milk in PBS for 1 hour at room temperature and incubated with the respective primary antibodies in 1% BSA in PBS containing 1% Tween-20 (PBS-T) overnight at 4°C. After 3 washes with PBS-T, HRP-conjugated secondary antibodies were added to the membrane for 1 hour at room temperature. Protein bands were then visualized using SuperSignal West Pico Chemiluminescent Substrate (ThermoFisher).

### Duolink proximity ligation assay

Cells plated on 8-well slides (40,000 cells/well) or frozen human liver slides were fixed and blocked as above. Respective combinations of antibodies from different species were used for detection. The Duolink proximity ligation assay (PLA) was performed according to the protocol provided by Sigma-Aldrich. After incubation with the primary antibody at 4°C overnight, the slides were washed 3 times with PBS-T and incubated with the respective combinations of PLA secondary antibodies for 1 hour at 37°C. Then the slides were washed twice with wash buffer A and incubated with ligation buffer for 30 minutes at 37°C. After another 2 washes with wash buffer A, the slides were incubated for 100 minutes at 37°C with amplification buffer. The reaction was stopped by 2 washes with wash buffer B and 1 wash with 0.01 X wash buffer B. The slides were then mounted with Duolink *In Situ* Mounting Medium with DAPI and pictures were taken the next day.

## Results

### Generation and characterization of different tagged OATP1B3

**Fig 1:**
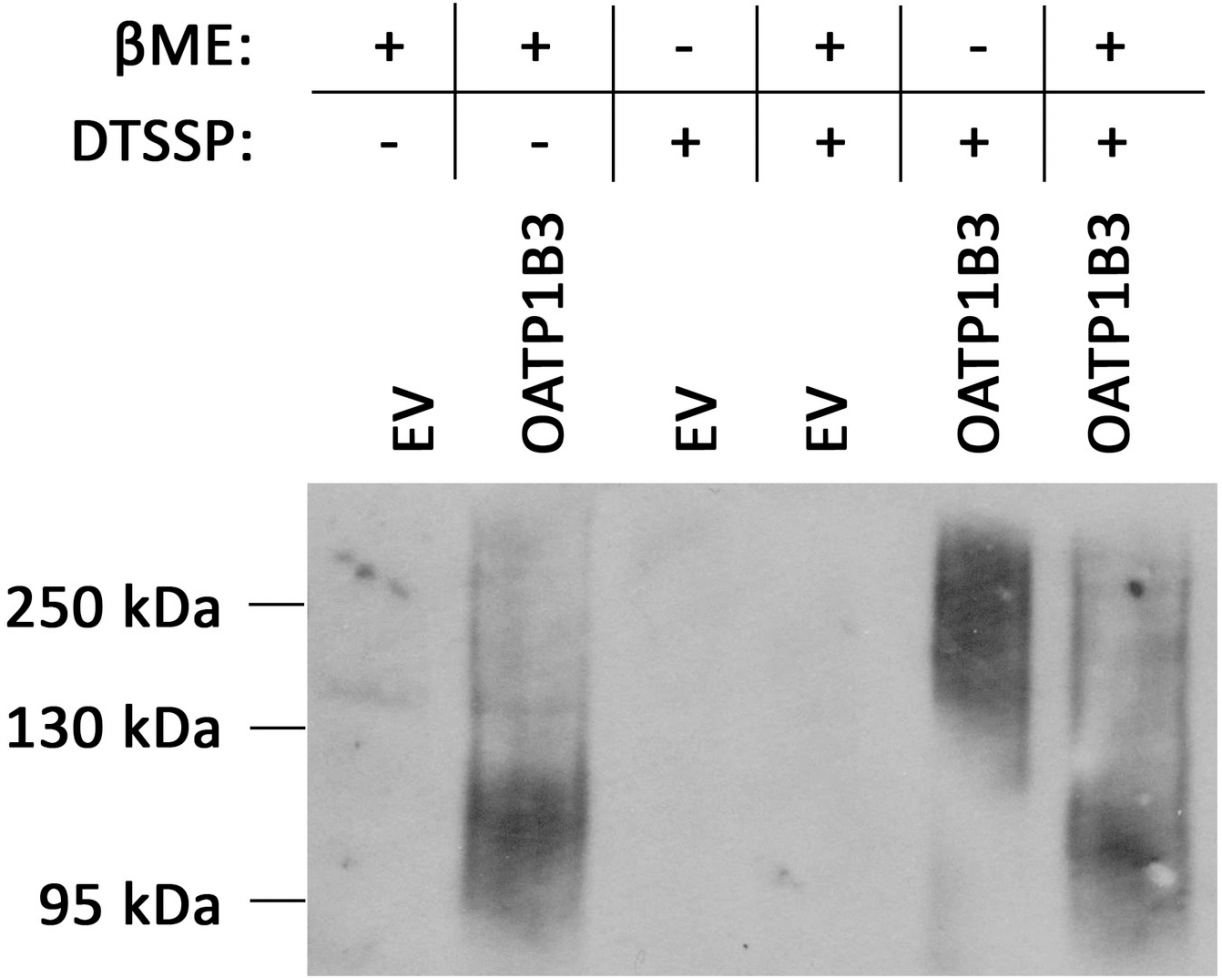
Western blot of His-tagged OATP1B3. HEK293 cells transfected with empty vector (EV) or His-tagged OATP1B3 were treated with or without DTSSP. Whole cell lysates were separated on a 7.5% gel in the presence or absence of β-mercaptoethanol (βME). After transfer to a PVDF membrane, His-tagged OATP1B3 was detected with an anti-His antibody.

**Fig 2:**
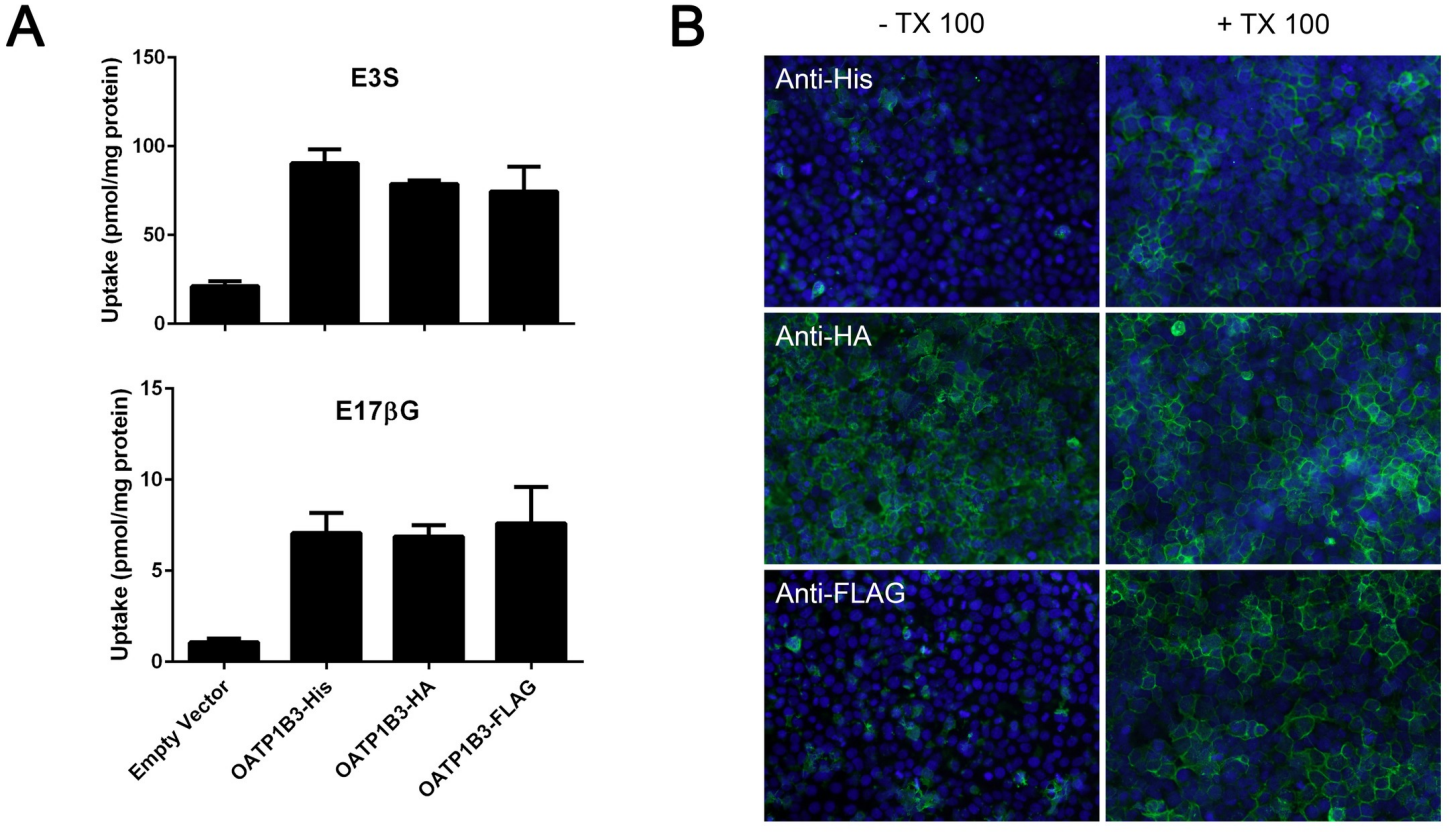
Characterization of differently tagged OATP1B3 proteins. (A) Uptake of 10 μM estrone-3-sulfate (E3S) or 1 μM estradiol-17-β-glucuronide (E17βG) was measured at 37°C with HEK293 cells transiently transfected with empty vector or OATP1B3 with a His-, HA- or FLAG-tag for 1 minute. Values are means ± SD of three independent experiments each performed with triplicate measurements. (B) HEK293 cells transiently transfected with OATP1B3 with a His-, HA- or FLAG-tag were fixed in the absence or presence of 1% TX-100. The different OATP1B3s were detected using the respective antibodies (shown in green) and nuclei were stained by DAPI (shown in blue).

Previous results from our lab showed that two bands were detected when OATP1B3 transiently expressed in HEK293 cells was analyzed on western blots; one at around 120kDa (normal size of OATP1B3) and another one at around 250kDa [22]. When the samples were treated with DTSSP, a crosslinker that contains a cleavable disulfide bond, the major band for OATP1B3 appeared around 250kDA, but returned to about 120kDa in the presence of β-mercaptoethanol (Fig 1). These results suggested that OATP1B3 could form dimers, either with itself or with other proteins. We first wanted to test the hypothesis that OATP1B3 could form homodimers and performed co-immunoprecipitation experiments using OATP1B3 constructs with different C-terminal tags. In addition to the already published OATP1B3-His [22], we also constructed OATP1B3-HA and OATP1B3-FLAG and characterized these constructs. Uptake of two model substrates, estradiol-17β-glucuronide (E17βG) and estrone-3-sulfate (E3S) was compared to OATP1B3-His which has the same function as wild type OATP1B3 [22]. As can be seen in Fig 2A all three constructs mediated uptake of both model substrates to the same extent, suggesting that the newly constructed HA- and FLAG-tagged OATP1B3 have the same function as wild-type and His-tagged OATP1B3. In order to test whether the commercial antibodies against the different tags would work for immunofluorescence and for the Proximity Ligations Assay (PLA) we first tested them using transiently transfected HEK293 cells. Under normal conditions, the C-terminal end of OATP1B3 is cytoplasmic [25] and an antibody reacting with the C-terminal end only stains cells if they have been permeabilized, e.g. with 1% Triton X-100. However, adding a tag of 6 to 9 amino acids with additional charges might change the topology of OATP1B3, at least at the C-terminal end. Therefore, we transiently transfected HEK293 cells with the three differently tagged OATP1B3 constructs and after 48 hours, incubated the fixed HEK293 cells in the absence and presence of 1% Triton X-100 before adding the respective antibodies. The results shown in Fig 2B demonstrate that the anti-His and anti-FLAG antibodies indeed only bound to transfected OATP1B3 after permeabilization with Triton X-100. However, the anti-HA antibody also resulted in positive staining in the absence of Triton X-100, suggesting that the C-terminal end of OATP1B3-HA is located on the extracellular side of the plasma membrane. None of the three antibodies used reacted with empty vector transfected HEK293 cells in the absence or presence of Triton X-100 (S1 Fig).

### Homo-oligomerization of OATP1B3

**Fig 3:**
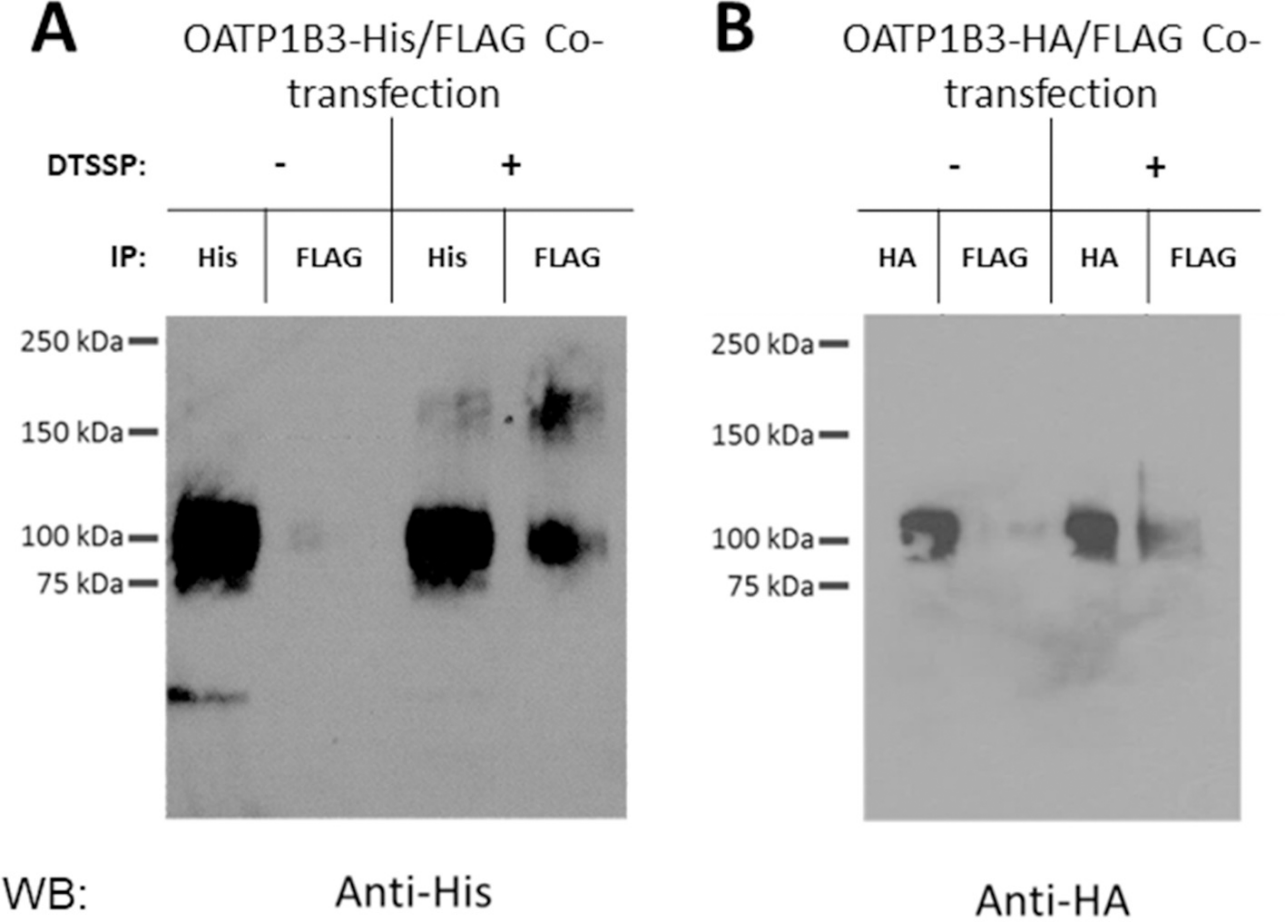
Immunoprecipitation of differently tagged OATP1B3 proteins. Membrane fractions isolated from HEK293 cells transiently transfected with OATP1B3-His and OATP1B3-FLAG (A) or OATP1B3-HA and OATP1B3-FLAG (B) and treated with or without DTSSP were solubilized. Immunoprecipitation was performed with Anti-His or Anti-FLAG antibodies (A), or Anti-HA or Anti-FLAG antibodies (B). The eluted proteins were detected with Anti-His antibody (A) or Anti-HA antibody (B).

To investigate whether OATP1B3 would be able to interact with itself, we transfected HEK293 cells with equal amounts of His- and FLAG-tagged OATP1B3 and 48 hours later we immunoprecipitated the respective OATP1B3 with either an anti-His or an anti-FLAG antibody. As can be seen in Fig 3A, immunoprecipitation with the anti-His antibody followed by western blotting with an anti-His antibody results in the detection of the His-tagged OATP1B3. Immunoprecipitation with the anti-FLAG antibody followed by western blotting with the anti-His antibody also allowed detection of His-tagged, and therefore co-immunoprecipitated OATP1B3. The obtained signals were much stronger after treatment with the cleavable chemical crosslinker DTSSP (Fig 3A, right hand side). Because the proteins were incubated in the presence of β-mercaptoethanol, which cleaves DTSSP, prior to gel separation, most of the immunoprecipitated proteins are seen at the molecular weight of the monomer. When the experiments were repeated with HA- and FLAG-tagged OATP1B3 and the western blot was probed with an HA-antibody a similar result was obtained (Fig 3B); immunoprecipitation with the HA-antibody also co-precipitated the FLAG-tagged OATP1B3. Thus, these results demonstrate that OATP1B3 can form homo-oligomers when expressed in HEK293 cells.

**Fig 4:**
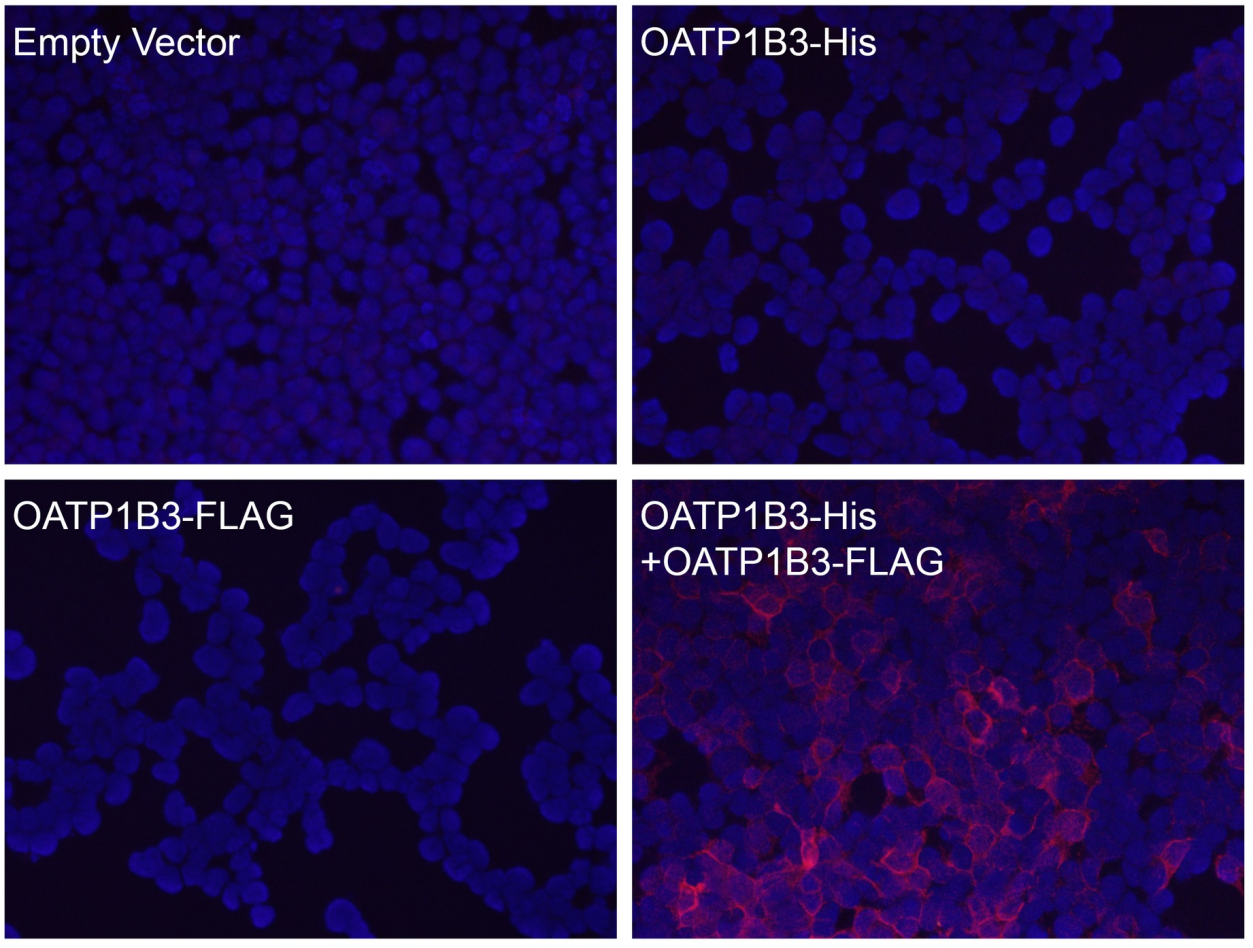
DuoLink proximity ligation assay (PLA) of OATP1B3-His and OATP1B3-FLAG. HEK293 cells were transiently transfected with empty vector, OATP1B3-His, OATP1B3-FLAG or both OATP1B3-His and OATP1B3-FLAG. After fixation, Duolink PLA was performed on all cells using a rabbit anti-His antibody in combination with a mouse anti-FLAG antibody. Co-localization signals are shown in red and nuclei in blue.

To confirm the co-immunoprecipitation results with an additional experimental approach and to demonstrate that OATP1B3 interacts with itself in the plasma membrane we used the Duolink PLA. In this assay, the interaction or close proximity (<40 nm) of two proteins, here OATP1B3-His and OATP1B3-FLAG or OATP1B3-FLAG with another His-tagged transporter (see below), is detected by using the respective anti-His and anti-FLAG antibodies that were raised in different species (rabbit anti-His and mouse anti-FLAG). After incubation with species specific secondary antibodies that have a short DNA sequence attached, the samples are incubated with linker DNA and a ligase to form DNA circles. Subsequently these circles are amplified in the presence of fluorescent nucleotides and the signals are detected using fluorescence microscopy. In Fig 4, a rabbit anti-His antibody and a mouse anti-FLAG antibody were used to perform PLA. Transfection of His- or FLAG-tagged OATP1B3 alone did not result in any signal, demonstrating that there was no cross-reaction between the mouse and rabbit PLA probe. However, when OATP1B3-His and OATP1B3-FLAG were co-transfected, clear signals at the plasma membrane could be detected, which confirmed the immunoprecipitation results and further demonstrated that OATP1B3 can form homodimers or oligomers when expressed in HEK293 cells.

### Functional influence of OATP1B3 oligomerization

**Fig 5:**
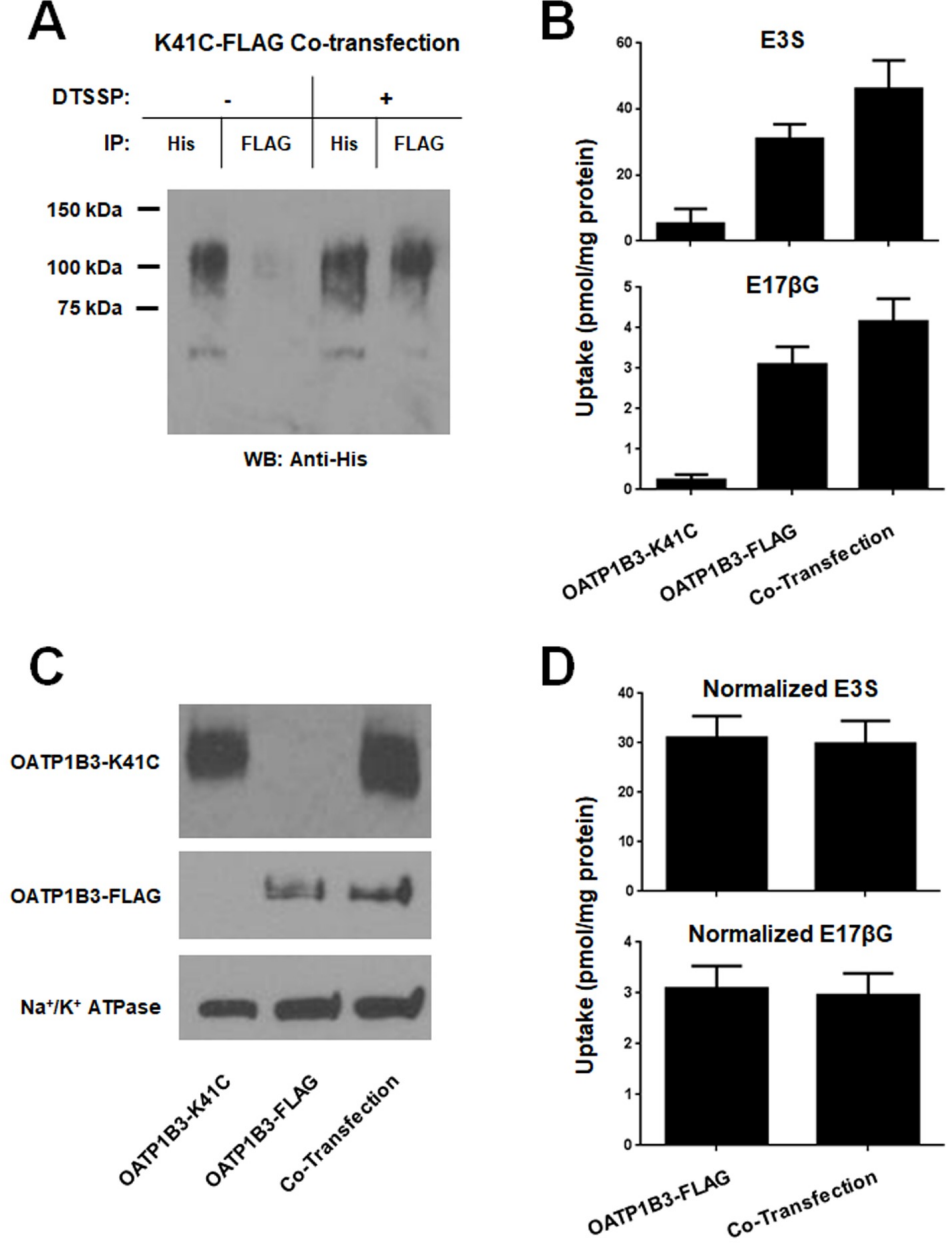
Effect of co-transfection of a-non-functional OATP1B3 mutant on function and oligomerization. (A) Membrane fractions isolated from HEK293 cells transiently transfected with OATP1B3-FLAG and OATP1B3-K41C-His and treated with or without DTSSP were solubilized. Immunoprecipitation was performed with Anti-His or Anti-FLAG antibodies and the eluted proteins were detected with Anti-His antibody. (B) HEK293 cells were transiently transfected with 500ng of OATP1B3-K41C-His plus 500ng of pcDNA5/FRT (Mutant), with 500ng of OATP1B3-FLAG plus 500ng of pcDNA5/FRT (WT), or with 500ng of OATP1B3-FLAG plus 500ng of OATP1B3-K41C-His (Co-Transfection). Forty-eight hours later uptake of 10μM E3S or 1μM E17βG was performed at 37°C for 1 min. (C) Surface biotinylated proteins were isolated from HEK293 cells transiently transfected as in (B). Western blot was performed with Anti-His (Mutant-His) or Anti-FLAG (WT-FLAG). Na^+^/K^+^ ATPase was used as loading control. (D) Uptake shown in (B) was normalized by the surface expressed mutant-His or WT-FLAG protein determined in (C) after subtracting of empty-vector control uptake.

Since there was abundant interaction of OATP1B3 at the plasma membrane, we further investigated whether oligomerization would affect transport activity. We used a non-transporting mutant of OATP1B3, OATP1B3-K41C-His, which contains a lysine at position 41 in place of the wild-type cysteine. To ascertain whether the mutant OATP1B3 is still capable of forming oligomers, we repeated the co-immunoprecipitation experiment and co-transfected the His-tagged mutant with wild-type OATP1B3-FLAG. The results in Fig 5A demonstrate that immunoprecipitation of the His-tagged mutant OATP1B3-K41C co-precipitated wild-type FLAG-tagged OATP1B3, especially after crosslinking with DTSSP. We then measured uptake of the two model substrates E3S and E17βG after co-transfection and compared the results with transport obtained with single transfections of either wild-type OATP1B3-FLAG or the non-functional mutant OATP1B3-K41C-His (Fig 5B). If OATP1B3 oligomers only work as a functional unit, and thus require two or more functionally active monomers, then the introduction of the mutant OATP1B3-K41C should abolish the whole unit function and there would be less uptake, if any. However, uptake into HEK293 cells that were transfected with 500ng of OATP1B3-FLAG plus 500ng of OATP1B3-K41C-His (co-transfection) was 25–35% higher than uptake into HEK293 cells transfected with 500ng of OATP1B1-FLAG plus 500ng of empty vector (OATP1B3-FLAG). Uptake into HEK293 cells transfected with 500ng of OATP1B3-K41C-His plus 500ng of empty vector (OATP1B3-K41C) was, as expected, very low. This result indicates that each OATP1B3 in the dimer or multimer can transport on its own. However, the question remained: why was transport higher after co-transfection as compared to single transfection? To answer this, we transfected HEK293 cells with increasing amounts of OATP1B3 plasmid (up to 1,000 ng per well) and measured uptake. The results demonstrated that uptake after transfection with 1,000 ng was more than twice the uptake after transfection with 500ng of plasmid (S2 Fig), suggesting that the amount of OATP1B3 at the plasma membrane was affected by differing the concentration of transfected plasmid. Therefore, we used surface biotinylation to determine the relative amounts of wild-type OATP1B3-FLAG and mutant OATP1B3-K41C-His at the plasma membrane after individual or co-transfection. The results shown in Fig 5C demonstrate that after co-transfection, indeed more OATP1B3 protein was detectable at the plasma membrane, explaining the increased uptake. After correcting for this increased amount of protein expressed at the membrane (OATP1B3 at the membrane after single transfection was set to 100% after correction with Na^+^/K^+^ ATPase), uptake was the same for the single transfection (wild-type OATP1B3-FLAG only) or the co-transfection with the nonfunctional mutant OATP1B3-K41C-His (co-transfection) (Fig 5D). To investigate whether dimerization/oligomerization with the mutant OATP1B3 would affect transport kinetics, we performed concentration-dependent uptake measurements on co-transfected cells using E17βG in the absence and presence of clotrimazole, a known OATP1B3 stimulator. As can be seen in Table 1, there was no difference in the K_m_ values for the monomer as compared to the dimer/oligomer. We would have expected that the V_max_ value would be decreased by about 50% because only half of the OATP1B3 constructs transfected were functional. However, the uptake was decreased by only 40%, which might be due to the small and statistically insignificant signal seen with the mutant alone (Fig 5B). In addition, similar to our previous report [12], the addition of clotrimazole increased the affinity for both the monomer as well as for the dimer/oligomer, suggesting that the allosteric stimulation is not affected by the dimer/oligomer formation and that OATP1B3 functions as two or more “monomers”.

**Table 1:** Effect of clotrimazole on kinetics of wild-type OATP1B3 in the absence and presence of the non-transporting OATP1B3-K41C.

Transporter	E17βG			E17βG + 30μM clotrimazole		
	Km (μM)	Vmax (pmol/mg protein x min^-1^)		Km (μM)		Vmax (pmol/mg protein x min^-1^)
WT-OATP1B3	7.1 ± 0.9	78 ± 3.3		3.3 ± 1.0		107 ± 8.3
WT-OATP1B3 + OATP1B3-K41C	5.5 ± 1.7	48 ± 4.5		3.6 ± 1.3		68 ± 6.5

HEK293 cells were transfected with 500ng of His-tagged OATP1B3 (WT-OATP1B3) or 250ng of His-tagged OATP1B3 plus 250ng of His-tagged OATP1B3-K41C. Kinetic parameters were determined based on the 30 secs uptake of 1, 5, 10, 20, 30 and 50μM E17βG in the absence or presence of 30μM clotrimazole.

### Hetero-oligomerization between OATP1B3 and other transporters

**Fig 6:**
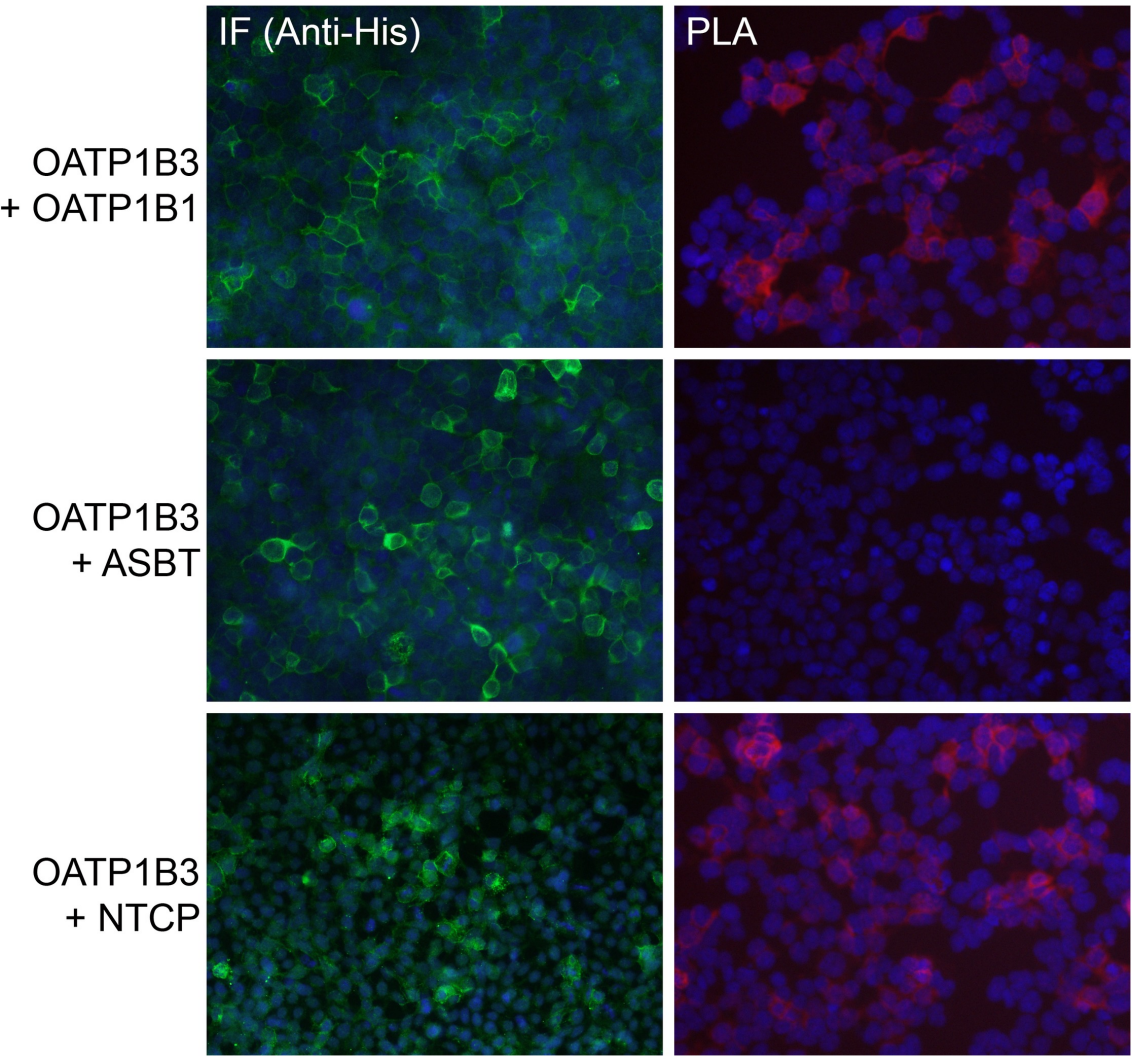
DuoLink PLA of OATP1B3-FLAG and other His-tagged transporters expressed in HEK293 cells. HEK293 cells were transiently transfected with His-tagged OATP1B1, ASBT or NTCP only or with these His-tagged transporters plus OATP1B3-FLAG. After 48 hours, cells were fixed and processed for immunofluorescence (IF) (signals in green) or Duolink PLA (signals in red). Nuclear staining is shown in blue.

Given that OATP1B3 can form homodimers/oligomers, we questioned whether OATP1B3 could interact with other transporters also expressed in the basolateral membrane of human hepatocytes, such as OATP1B1 and NTCP. We performed Duolink PLA experiments using HEK293 cells that were transiently transfected with OATP1B3-FLAG and His-tagged OATP1B1 or NTCP. As a negative control, we used His-tagged ASBT, another bile acid transporter, which is not expressed in human hepatocytes. As shown in Fig 6 on the left side, all the His-tagged proteins were expressed and could be detected by immunofluorescence (IF) with an anti-His antibody. Fig 6 also demonstrates (right side) that the co-expression of OATP1B3 with either OATP1B1 or with NTCP resulted in good PLA signals at the plasma membrane, suggesting the formation of hetero-oligomers of the two respective proteins. Importantly, the fact that co-expression of OATP1B3 with ASBT did not result in any signals demonstrates that the proximity of the proteins that yielded positive signals was not just due to overexpression of the transporters in HEK293 cells.

**Fig 7:**
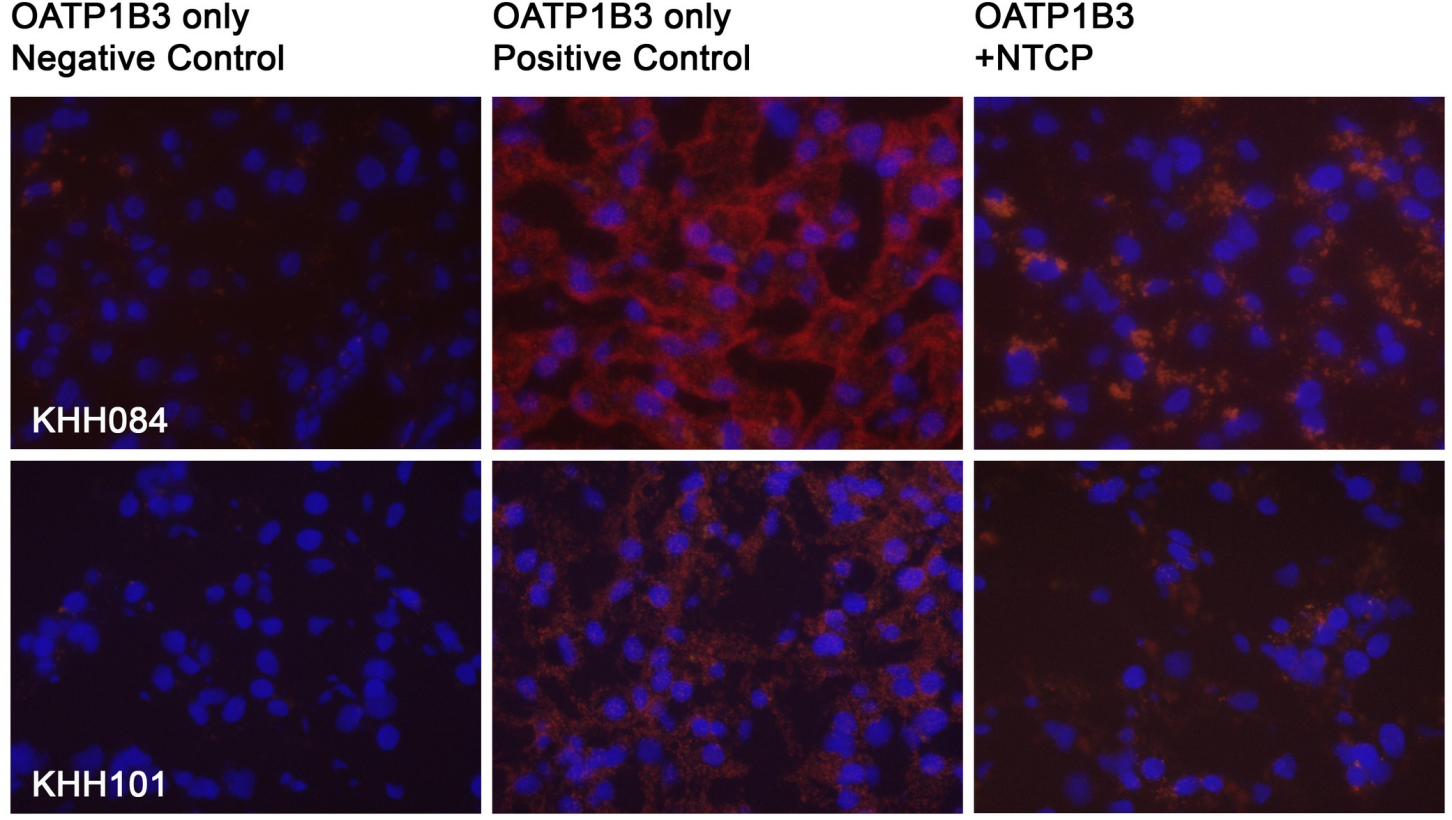
Co-localization of OATP1B3 with NTCP in human liver. Frozen human liver sections were used to perform Duolink PLA. For the OATP1B3 negative control an anti-rabbit minus and an anti-mouse plus PLA antibody were used; for the OATP1B3 positive control, an anti-rabbit minus was combined with an anti-rabbit plus PLA antibody. To co-localize OATP1B3 with NTCP, a goat anti-NTCP antibody was combined with the rabbit anti-OATP1B3 antibody. Co-localization is shown in red, nuclei are stained with DAPI and are shown in blue. KHH084: 59 year old male; KHH101: 57 year old female.

To further confirm that OATP1B3 and NTCP also interact in human liver and not only in transfected HEK293 cells, we performed PLA experiments using frozen human liver tissues. Fig 7 shows that compared to the OATP1B3 negative control, positive PLA signals were obtained for the OATP1B3 positive control as well as for the OATP1B3-NTCP interaction, confirming that OATP1B3 and NTCP also interact in human liver.

## Discussion

In this study, we demonstrated that the important hepatocellular drug transporter OATP1B3 can form homo-dimers or -oligomers. Within these dimers/oligomers, an individual OATP1B3 seems to be the functional unit because co-transfections with a nonfunctional OATP1B3-K41C mutant did not affect uptake of E3S and E17βG. Furthermore, we also established that OATP1B3 can form hetero-dimers/oligomers in HEK293 cells with other hepatocellular transporters like OATP1B1 and NTCP, and we verified the interaction with NTCP using frozen human liver sections.

Several lines of evidence support our findings. Co-immunoprecipitation experiments confirmed a physical interaction between OATP1B3-His and OATP1B3-FLAG (Fig 3), while the Proximity Ligation Assay verified a close association of the two differently tagged OATP1B3 proteins (Fig 4). In addition, our data also show that the C-terminal end of OATP1B3 is not involved in dimerization/oligomerization since both His-tagged OATP1B3, with the C-terminal end on the cytoplasmic side of the membrane, as well as HA-tagged OATP1B3, with the C-terminal end on the extracellular side of the membrane (Fig 2B), were able to co-immunoprecipitate with FLAG-tagged OATP1B3 (Fig 3B).

At the functional level, our results show that a non-functional unit (OATP1B3-K41C) in the homo-dimer/oligomer does not affect normal substrate transport by OATP1B3, nor does it affect the modulation of transport by clotrimazole. These results suggest that each unit within the dimer/oligomer works as an independent functional unit, which is similar to previous findings regarding NTCP [19]. However, our co-transfection studies with the non-functional mutant did not yield any structural information with respect to which amino acid residues or transmembrane domains are potentially involved in the interaction(s). A recent report suggested that GXXXG motifs present in OATP1B1 are involved in the oligomerization of OATP1B1 [21]. The three GXXXG motifs in OATP1B1 are completely conserved in OATP1B3 and therefore, it is possible that G393 in the third GXXXG motif is involved in homo-oligomerization. Given that there are also three GXXXG motifs in NTCP, it is possible that these motifs also play a role in hetero-oligomerization or that they are important in intramolecular interactions and stabilize proper folding [26]. To evaluate these possibilities future experiments are required, including, for example, mutational analysis, where the glycine residues are replaced by other amino acid residues (besides alanine) to determine the effect these mutations have on plasma membrane localization of the proteins.

Using the proximity ligation assay in transiently transfected HEK293 cells, we also demonstrated that OATP1B3 interacts with OATP1B1 and with NTCP, two additional transporters expressed at the basolateral membrane of human hepatocytes. Furthermore, we confirmed the interaction between OATP1B3 and NTCP on frozen human liver sections. The functional consequences of these interactions will also have to be evaluated in future studies. The proximity ligation assay only demonstrates that two proteins are within 40nm of each other, but does not prove a direct physical interaction between the two proteins. Thus, additional experiments will be required to investigate whether the identified interaction partners, OATP1B1 and NTCP, are directly interacting with OATP1B3 or whether they reside together in the same membrane micro-domains. Assuming that these interactions between the two different transport proteins are direct protein-protein interactions, we can speculate that the two proteins might affect each other’s function. This could have consequences for potential drug-drug interactions studies (see below). In contrast, if the two proteins do not directly interact and are just expressed in the same micro-domains, then OATP1B3 and NTCP might be regulated in a similar manner, at least at the post-translational level. Again, additional experiments are required to characterize these possible mechanisms in detail.

Previous western blot data has suggested homo- or hetero-dimerization/oligomerization for OATP1B3 [22] and OATP1B1 [27], as well as other solute carriers, including ASBT [28], Na^+^-K^+^-2Cl^-^ cotransporter (NKCC1) [29], SLC10A5 [30], and SLC10A7 [31]. Furthermore, it was demonstrated that NTCP can homo- and heterodimerize with other members of the SLC10A family [19]. Thus, it is very likely that many solute carriers work as functional dimers/oligomers and could potentially be located in the same membrane micro-domains.

The fact that OATP1B3 can hetero-oligomerize with other transporters suggests that these transporters potentially influence each other’s function and might even be regulated in a similar manner at the protein level.

Most OATPs show increased function at an acidic pH value [32, 33]. While this seems to be an advantage for an intestinal transporter one could argue that it should not affect a transporter expressed at the basolateral membrane of hepatocytes. However, co-localization in membrane micro-domains or direct interactions with a Na^+^/H^+^-exchanger could lead to an acidic microenvironment which could increase OATP function. Furthermore, if the microenvironment generated around these transporter micro-domains indeed influences the physiological or pharmacological function of the involved drug transporters, drug-drug interaction studies should be performed with cell lines that express multiple transporters rather than just a single transporter. Recent findings involving members of the cytochrome P450 family of drug metabolizing enzymes suggest that in vitro systems with more than a single transporter are not only more physiologically relevant, but might indeed demonstrate that that the different transporters affect each other’s function. The activity of CYP2C9 was modified by co-expression with different amounts of CYP3A4 [34]. In analogy, OATP1B3 activity could be modulated by any of the interacting transporters and vice versa. As a consequence, the functional importance of OATP1B3 interaction with other transporters will have to be evaluated.

In summary, we have shown that the multi-specific liver transporter OATP1B3 can form homo-oligomers and can interact with other transporters in the liver, including OATP1B1 and NTCP. We also demonstrated that the homo-oligomers work as two or more individual functional subunits. Our findings are important because they suggest the existence of transporter micro-domains containing OATPs, and as a consequence, functional transporter studies might have to be modified to include co-expression systems in the future.

## Supporting information

S1 Fig Immunofluorescence control of the three antibodies used to detect His-, HA- and FLAG-tagged OATP1B3. HEK293 cells transiently transfected with empty vector were fixed in the absence or presence of 1% TX-100. The cells were then incubated with either anti-His, anti-HA or anti-FLAG antibodies followed by the respective secondary antibodies that should result in a green signal. Nuclei were stained by DAPI (shown in blue).
(DOCX)

S2 Fig Effect of co-transfection of different ratios of empty vector and OATP1B3-FLAG. Uptake of 1 μM estradiol-17β-glucuronide (E17βG) was measured at 37°C with HEK293 cells transiently transfected with a total amount of 1000 ng of cDNA for 1 minute. Mean ± SD of The percentage of OATP1B3-FLAG cDNA is indicated on the x-axis.
(DOCX)
