# Supplementary material for: Organic anion transporting polypeptide 1B3 can form homo- and hetero-oligomers
Source: PLoS One. 2017 Jun 23;12(6):e0180257. doi: 10.1371/journal.pone.0180257 (PMC5482489; doi:10.1371/journal.pone.0180257)
Supplement: S1 Fig — HEK293 cells transiently transfected with empty vector were fixed in the absence or presence of 1% TX-100. The cells were then incubated with either anti-His, anti-HA or anti-FLAG antibodies followed by the respective secondary antibodies that should result in a green signal. Nuclei were stained by DAPI (shown in blue). (DOCX) [file pone.0180257.s001.docx]

**S1 Fig.** **Immunofluorescence control of the three antibodies used to detect His-, HA- and FLAG-tagged OATP1B3.
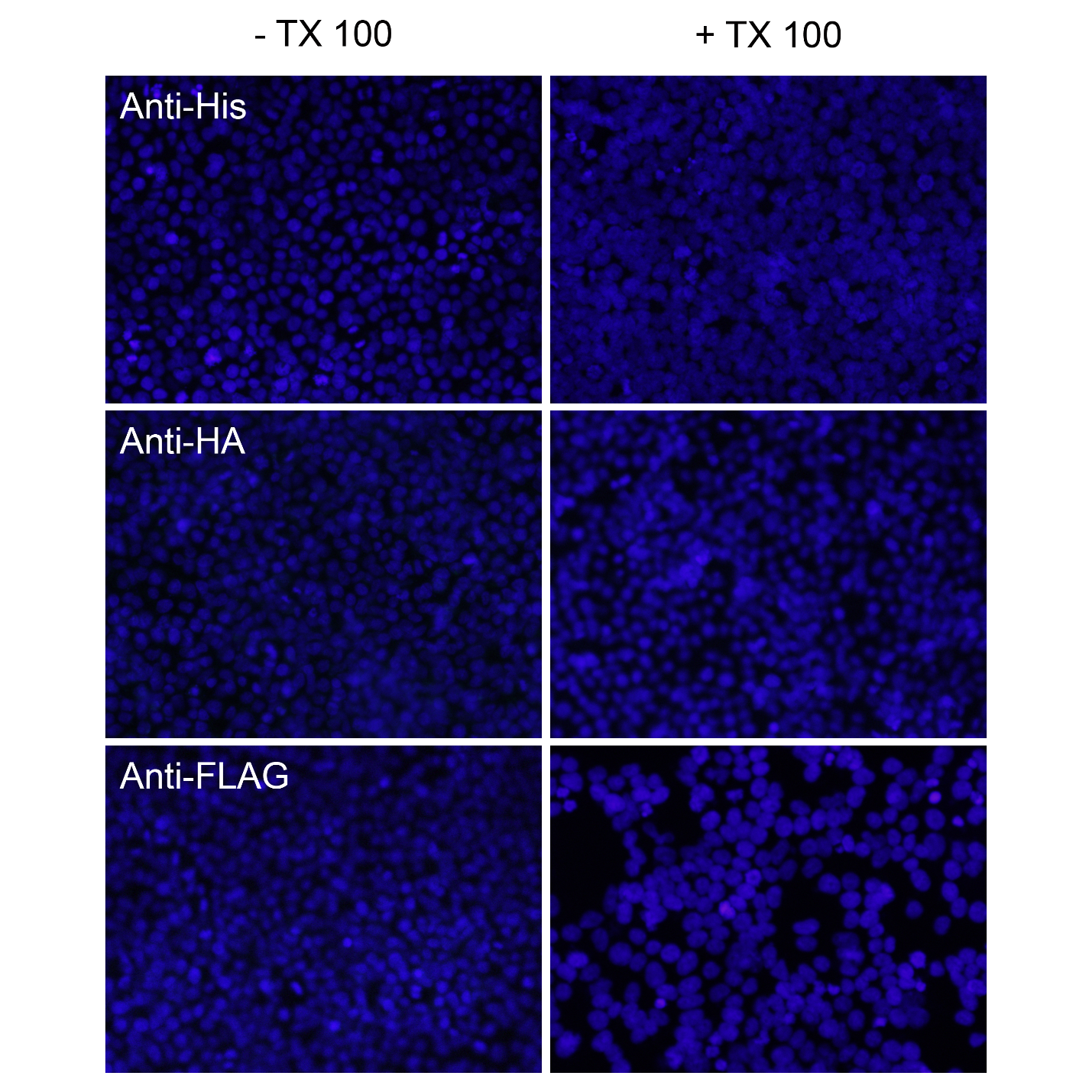
**

HEK293 cells transiently transfected with empty vector were fixed in the absence or presence of 1% TX-100. The cells were then incubated with either anti-His, anti-HA or anti-FLAG antibodies followed by the respective secondary antibodies that should result in a green signal. Nuclei were stained by DAPI (shown in blue).
