# Supplementary material for: Organic anion transporting polypeptide 1B3 can form homo- and hetero-oligomers
Source: PLoS One. 2017 Jun 23;12(6):e0180257. doi: 10.1371/journal.pone.0180257 (PMC5482489; doi:10.1371/journal.pone.0180257)
Supplement: S2 Fig — Uptake of 1 μM estradiol-17β-glucuronide (E17βG) was measured at 37°C with HEK293 cells transiently transfected with a total amount of 1000 ng of cDNA for 1 minute. Mean ± SD of The percentage of OATP1B3-FLAG cDNA is indicated on the x-axis. (DOCX) [file pone.0180257.s002.docx]

**S2 Fig.** **Effect of co-transfection of different ratios of empty vector and OATP1B3-FLAG.**

Uptake of 1 µM estradiol-17β-glucuronide (E17βG) was measured at 37°C with HEK293 cells transiently transfected with a total amount of 1000 ng of cDNA for 1 minute. Mean ± SD of The percentage of OATP1B3-FLAG cDNA is indicated on the x-axis.
